# A digital workflow for pair matching of maxillary anterior teeth using a 3D segmentation technique for esthetic implant restorations

**DOI:** 10.1038/s41598-022-18652-4

**Published:** 2022-08-23

**Authors:** Jin-Woo Choi, Gyu-Jin Choi, Yu-Seong Kim, Min-Ho Kyung, Hee-Kyung Kim

**Affiliations:** 1 Department of Prosthodontics, Institute of Oral Health Science, Ajou University School of Medicine, Suwon, Republic of Korea; 2 Lifemedia Interdisciplinary Program, Ajou University, Suwon, Republic of Korea; 3 Department of Digital Media, Ajou University, Suwon, Republic of Korea

**Keywords:** Biotechnology, Computational biology and bioinformatics, Medical research, Mathematics and computing

## Abstract

We investigated a state-of-the-art algorithm for 3D reconstruction with a pair-matching technique, which enabled the fabrication of individualized implant restorations in the esthetic zone. This method compared 3D mirror images of crowns and emergence profiles between symmetric tooth pairs in the anterior maxilla using digital slicewise DICOM segmentation and the superimposition of STL data. With the outline extraction of each segment provided by 100 patients, the Hausdorff distance (HD) between two point sets was calculated to identify the similarity of the sets. By using HD thresholds as a pair matching criterion, the true positive rates of crowns were 100, 98, and 98%, while the false negative rates were 0, 2, and 2% for central incisors, lateral incisors, and canines, respectively, indicating high pair matching accuracy (> 99%) and sensitivity (> 98%). The true positive rates of emergence profiles were 99, 100, and 98%, while the false negative rates were 1, 0, and 2% for central incisors, lateral incisors, and canines, respectively, indicating high pair matching accuracy (> 99%) and sensitivity (> 98%). Therefore, digitally flipped contours of crown and emergence profiles can be successfully transferred for implant reconstruction in the maxillary anterior region to optimize esthetics and function.

## Introduction

Dental implants are becoming a popular and effective choice, in terms of the appearance and function of a natural tooth, to replace missing teeth. As patients' esthetic expectations continually increase, replacement of missing teeth with dental implants in the anterior maxilla is very challenging for clinicians. In an attempt to attain ideal esthetics, peri-implant soft and hard tissue architectures should be congruent with those of the surrounding adjacent teeth^1,2^. Optimizing the emergence profile, which is the contour of a restoration where it emerges from the gingiva, can be a key factor for improving clinical outcomes of implant restorations in the anterior maxilla. A proper emergence profile can give a smooth, natural-looking transition from the circular implant platform to the implant crown at the gingival level, resulting in a lifelike appearance^3–5^. In addition, the reproduction of an appropriate emergence profile can provide a favorable biological response to the surrounding soft tissue and, eventually, the underlying bone^6–8^.

Over the past decade, digital technology has developed rapidly in dentistry. With the introduction of novel digital devices and processing software, digital dentistry could provide new ways of obtaining a diagnosis, performing treatment planning, fabricating restorations, and communicating with patients. Significantly, the advent of three-dimensional (3D) digital scanners allows the creation of 3D models with the collected data on the surface of the subject. Nevertheless, several factors, such as the scanning technique (intraoral or extraoral)^9,10^, the type of substrate^11^, scanning distance^10,12^, and operator experience^11^, could affect the degree of image deviation although the use of digital scanners has been rapidly incorporated into everyday dental practice. A previous study^9^ showed larger dimensional shrinkage in precision measurements with a direct intraoral scanner compared to that determined with an extraoral laboratory scanner. Furthermore, digital matching of optical scanning data with cone-beam computed tomography (CBCT), which provides volumetric data on bone or tooth structures, has been performed to enhance the accuracy of implant placement. However, Komuro et al.^12^ suggested that dimensional shrinkage should be taken into account with regard to the reliability of the matching technique.

Currently, prosthetic dentistry is becoming increasingly personalized with the aid of digital workflows as a digital esthetic solution. Computer-aided design/computer-aided manufacturing (CAD/CAM) technology enables dental professionals to design customized implant superstructures^13,14^. In a situation where a hopeless tooth exists, a 3D surface mesh of the tooth structure would be saved before tooth extraction with an optical scanning device and the scanned data could be transferred and used as a template for the implant. In contrast, in an edentulous space that no longer has teeth, the patient-specific CAD design can be selected from the data libraries installed on a CAD/CAM system^15^. A mirror image of the contralateral tooth was also used to create an implant prosthetic design. A previous study^16^ reported that an individualized CAD/CAM healing abutment could be fabricated with a digitally flipped image of the opposite side.

Bilateral symmetry of human body structures relative to the mid-sagittal plane is assumed to be beneficial with respect to form and function from an evolutionary standpoint^17^. As with other subunits of the face, left/right symmetry of the dental components has been associated with balanced oral functions and the perception of facial beauty^18^. Symmetry has been considered normal in a healthy face and thus, any facial asymmetry may cause both physiological and psychological problems. In this regard, efforts have been made to quantitatively analyze the bilateral symmetric patterns of dental structures, such as root anatomy^19,20^, arch forms^21^, or orthodontic landmarks^22,23^ in a consistent way.

Bioesthetics in dentistry, which provide optimum dental health, comfort, beauty, and appearance, may include more than six maxillary anterior teeth. However, this study focused on the maxillary anterior dentition because they are important in achieving a beautiful smile and good function due to their positions^24^. In addition, anterior aesthetics are a key factor for evaluating transverse interarch spaces in orthodontic treatment^25^. When using a mirrored image of the contralateral tooth for implant reconstruction, it is important to ensure that a tooth from the opposite side of the same arch would be a plausible template for the pair-matching approach. However, there have been no studies investigating quantitative pair matching between bilateral teeth with the same notation. In this study, we identified true pairs of crown and emergence profile contours in the upper anterior dentition based on deep learning-based image extraction and 3D space comparison. Moreover, this study depicted an effective pair-matching algorithm for tooth shapes, including 3D image processing with dental CAD/CAM technologies. Therefore, the purpose of this study was to evaluate morphology-based quantitative pair matching for individual-specific implant restoration in the upper anterior dentition. The null hypothesis was that there would be no differences in 3D morphologies among contralateral tooth pairs in the anterior maxilla in terms of crown and emergence profiles.

## Results

The Shapiro‒Wilk test indicated that the variables had normal distributions (*p* > 0.05). With regard to the qualitative variables on the determination of specific dental structures, Cohen’s *k* value was 0.821 (95% confidence interval (95% CI) = 0.703–0.924), indicating a high interoperator agreement according to the Landis and Koch guidelines^26^.

**Figure 1:**
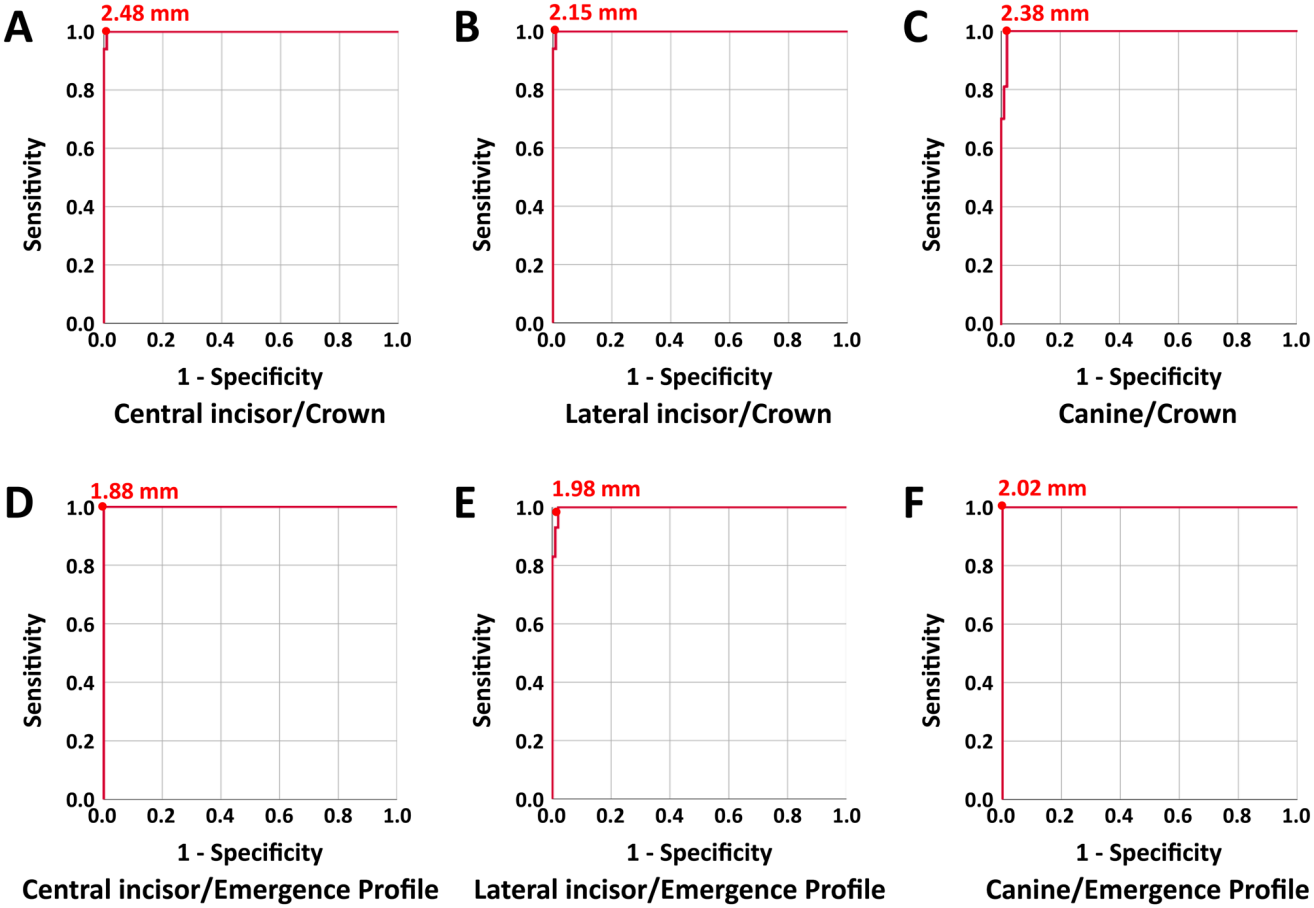
ROC curves for 3D HD thresholds for pair matching central incisors (**A**, crown; **D**, emergence profile), lateral incisors (**B**, crown; **E**, emergence profile), and canines (**C**, crown; **F**, emergence profile). The smallest HD (mm) for 100% sensitivity was marked.

As shown in the receiver operating characteristic (ROC) curves (Fig. 1), the smallest Hausdorff distance (HD) that provided 100% sensitivity for each segment was central incisor crown = 2.46 mm, central incisor emergence profile = 1.88 mm, lateral incisor crown = 2.15 mm, lateral incisor emergence profile = 1.98 mm, canine crown = 2.38 mm, and canine emergence profile = 2.02 mm.

**Figure 2:**
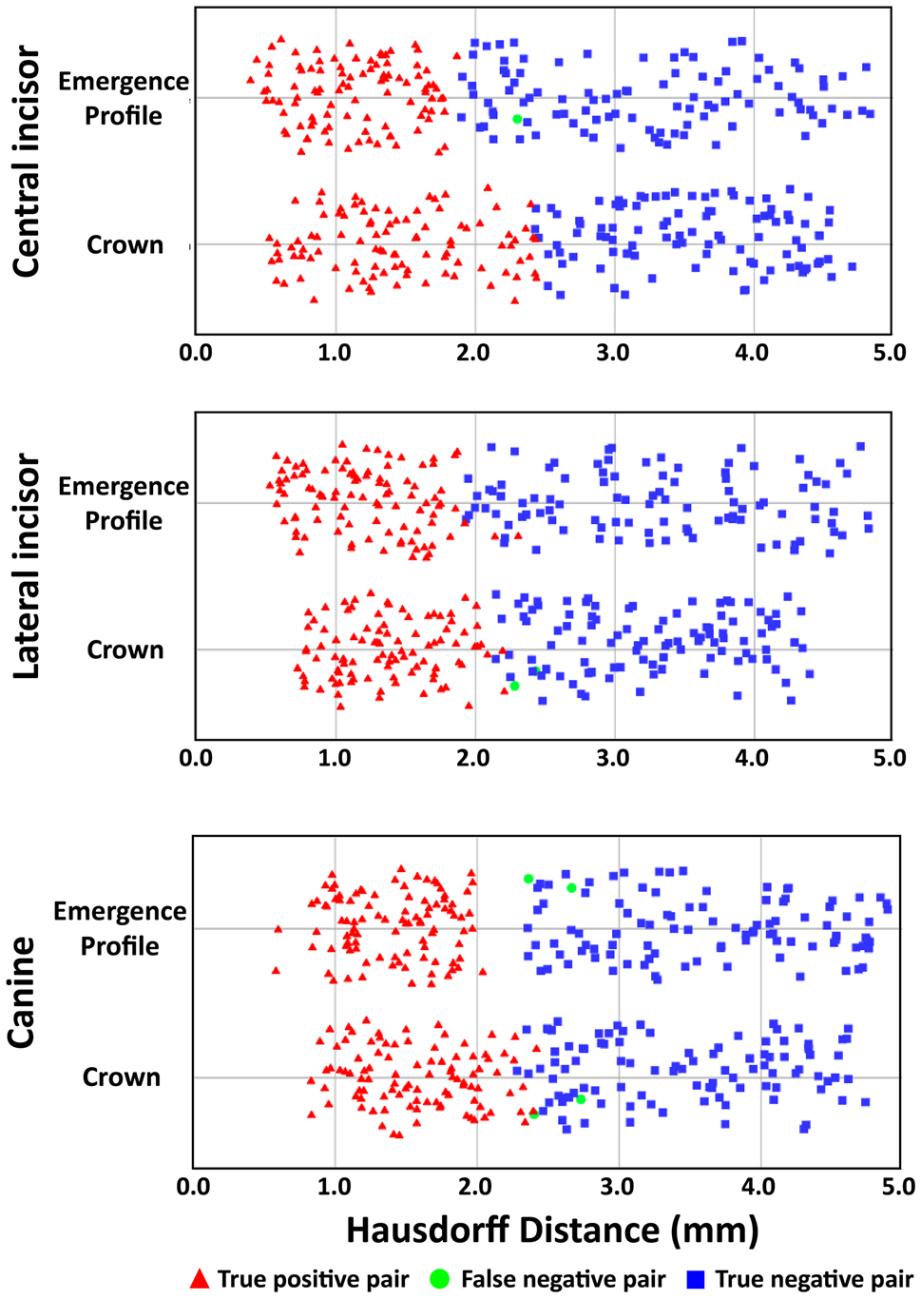
Jitter plot of 3D HDs for central incisor, lateral incisor, and canine pair comparisons. A true positive pair indicates a contralateral element, from the same individual, that was correctly categorized as a pair. For the distribution of true negative pairs, the smallest 100 data points among the 9900 comparisons were illustrated.

In this study, the minimum HD at 100% specificity was set as the threshold to define a match. The means of true positive pairs, false negative pairs, and true negative pairs were significantly different (*p* < 0.05) and their distributions are presented in Fig. 2. The central incisor crowns and lateral incisor emergence profiles had no false negative pairings.

By using HD thresholds as a pair matching criterion, the true positive rates of crowns were 100, 98, and 98%, while the false negative rates were 0, 2, and 2% for central incisors, lateral incisors, and canines, respectively, indicating high pair matching accuracy (> 99%) and sensitivity (> 98%). The true positive rates of emergence profiles were 99, 100, and 98%, while the false negative rates were 1, 0, and 2% for central incisors, lateral incisors, and canines, respectively, indicating high pair matching accuracy (> 99%) and sensitivity (> 98%). Table 1 presents the overall performance results based on the HD threshold criterion.

**Table 1:** Overall performance results (%) based on the HD thresholds criterion.

Element		Sensitivity	Accuracy
Central incisor	Crown	100.0	100.0
	Emergence profile	99.0	99.0
Lateral incisor	Crown	98.0	99.0
	Emergence profile	100.0	100.0
Canine	Crown	98.0	99.0
	Emergence profile	98.0	99.0

## Discussion

This study introduced a digital approach to contralateral pair matching for a customized design of esthetic implant restorations. Instead of full mesh-to-mesh comparisons, our workflow involved 3D shape segmentation of the specific elements (crown and emergence profiles) from the surrounding structures and we compared a portion of an image allocated to the region of interest (ROI). With this approach, we can reduce the average time cost. In addition, this study entailed a deep learning-based neural network architecture for ROI detection and segmentation, which can significantly enhance the accuracy of computer vision tasks. To improve the segmentation and generalization abilities of the network, we applied training-time augmentations, which consisted of randomized operations, such as cut-out, cut-paste, elastic deformation and rotation.

Although dental implants have become the best treatment option to replace missing teeth, they have some esthetic limitations, especially in the posterior region. Typically, an anterior tooth has a single root, while a posterior tooth has multiple roots with complex shape variations. As a substitute for a multirooted posterior tooth, it might be difficult to duplicate a natural emergence profile with a single root-form implant. The discrepancy in the anatomic dimensions between an implant and a posterior tooth could negatively affect the esthetic appearance of the final restoration. Felsypremila et al. study^27^ found that the anatomic symmetry of the root morphology of posterior teeth varied from 70 to 98% with maximum asymmetry in the maxillary second molar (29.2%). Therefore, we selected the maxillary anterior region in terms of a replication of the tooth structures with the reverse image of a contralateral tooth to achieve a predictable esthetic result.

Facial symmetry can greatly improve one’s appearance since facial esthetic subunits are highly sensitive to asymmetry. As seen within the results of a previous study^18^, the bilateral symmetry of facial structures did not imply that two objects in a mirrored pair exactly resembled each other. The differences in eyelid position of > 2 mm, oral commissure of > 3 mm, and brow position of > 3.5 mm were identified as asymmetric pairs^17^. Although the abovementioned study used digitally manipulated 2D models, there might bet a certain physiologic threshold across facial features that limits the asymmetry within bilateral pairs. This study proposed quantitative metrics to assess the similarity between a left/right tooth pair by computing the Hausdorff distances between 3D meshes. With respect to a threshold of asymmetry, we found a limited degree of discrepancy (up to 2.15–2.46 mm for crowns and up to 1.88–2.02 mm for emergence profiles) between bilateral symmetric tooth pairs in the upper anterior dentition.

The tooth position could be an important aspect as far as the symmetry and balance of the dental characteristics were concerned. Previous studies^28,29^ demonstrated that symmetry was less critical as the tooth position became farther from the midline, and thus, asymmetric canines would be more tolerable than other anterior teeth. In this study, we also observed that canine pairs had more false negative pairs than central incisor pairs and lateral incisor pairs, indicating the existence of minor asymmetry in the canine pairs.

According to the results of this study, high pair matching accuracy (> 99%) and sensitivity (> 98%) for crown elements and high pair matching accuracy (> 99%) and sensitivity (> 98%) for emergence profile elements were obtained. Therefore, the null hypothesis was accepted. These results support the practically feasible approach to designing a patient-specific implant restoration in an esthetically demanding area using a digitally flipped image of the contralateral tooth. The proper emergence profile and crown shapes can improve esthetic outcomes and biological responses of implant restorations.

However, there are limitations to this study that should be noted. During imaging, STL segmentation, and postprocessing, shape errors could be inevitable. With regard to computer-based image detection and analysis, properly categorized and annotated datasets would be a crucial factor in the medical diagnosis. In this study, the region of interest was specified with a freehand-drawn mask and thus, the manual annotation would negatively affect the accuracy of the image analysis and segmentation. The deep learning-based automatic annotation workflow^30^ can be used to enhance the accuracy and speed of the annotation process for further studies. In teeth with large interproximal contact areas, any uncertainty during the process of intersecting triangles for the connected component might affect the generation of STL data. The CBCT image quality may vary depending on several factors, such as the threshold of gray value, artifacts, and radiation doses. Although we tried to reduce the field of view (FOV) and the radiation dose with a controlled gray-value threshold, artifact reduction software^31^ was not applied in this study. The artifact reduction tool should be used to decrease artifacts and to obtain better quality CBCT images for further studies. In addition, a reduction in the STL data size (1.0 mm × 1.0 mm × 1.0 mm) might also affect subsequent operations. Future studies should investigate a data size reduction approach in controlling the accuracy of an image-processing algorithm for dental structures.

## Conclusion

We investigated a quantitative pair-matching algorithm by using digital technologies, and high pair-matching accuracy was obtained in the maxillary anterior region. The results of this study support the practically feasible approach to create implant superstructures using a digitally flipped image in the upper anterior dentition to improve aesthetics and function.

## Methods

### 3D laboratory scanning

**Figure 3:**
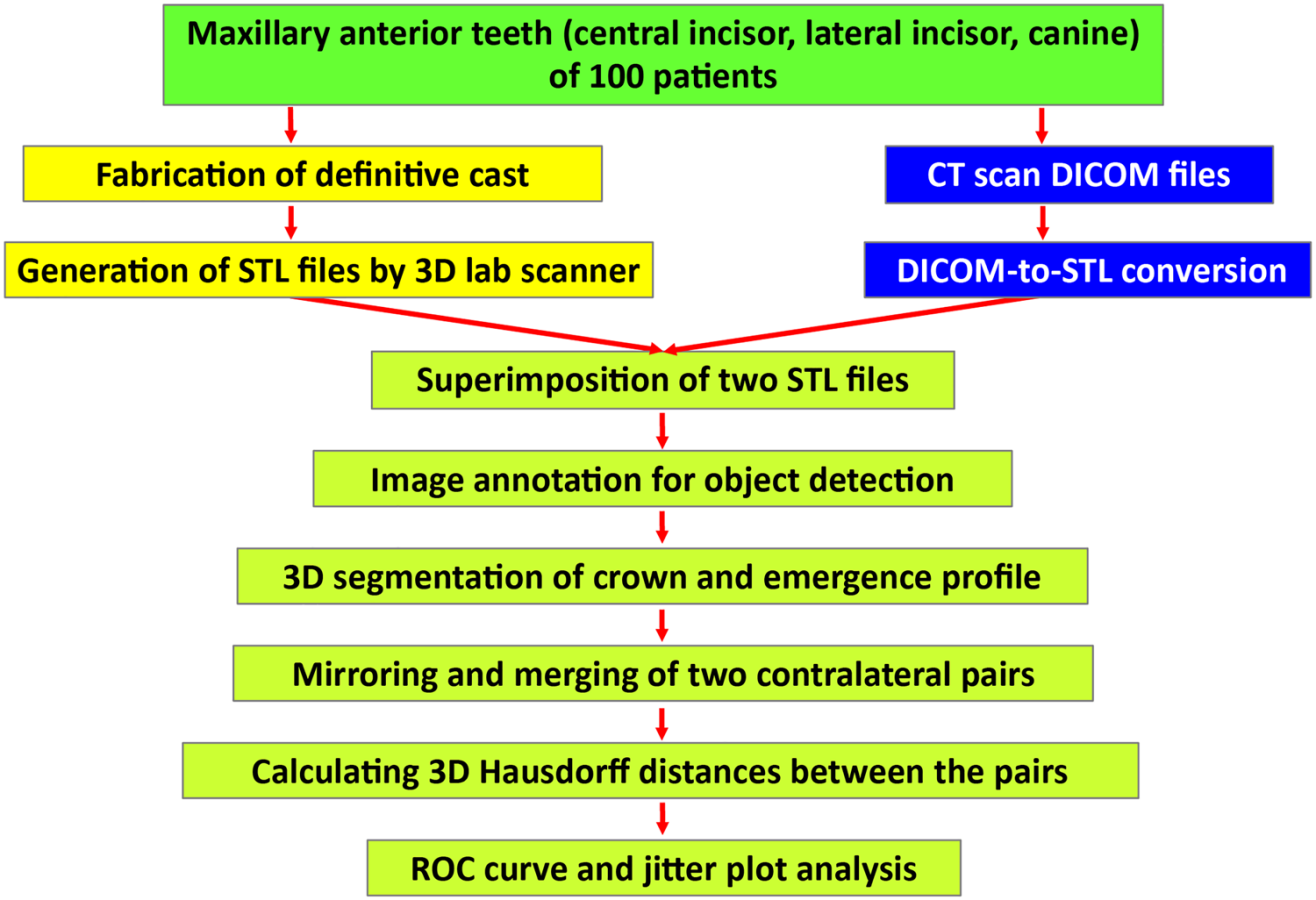
Summary of the workflow of the study process.

All experiments and methods were performed in accordance with relevant guidelines and regulations. All experimental protocols were approved by the Institutional Review Board (IRB) of Ajou University Hospital (no. AJIRB-MED-MDB-21-395), and written informed consent was obtained from all patients. In this study, the stone casts of 100 patients at Ajou University Hospital who had CBCT scans taken for various clinical indications were randomly selected in a double-blind manner to minimize selection bias. The summarized inclusion/exclusion criteria are presented in Table 2. Figure 3 outlines the digital workflow of contralateral pair matching in this study. We, the authors, are three dentists (clinical team) and two computer scientists (digital team). To enhance the accuracy and reliability of the measurements, device calibration and operator training were performed. The scanning procedure and the clinical measurements were executed by the calibrated clinical team, while the computational parts were carried out by the calibrated digital team. All operators were involved in the determination of specific periodontal parameters and the 3D image superimpositions. The definitive model was scanned using a desktop 3D laboratory scanner (E1; 3Shape, Copenhagen, Denmark) and the mesh was exported to an STL file.

**Table 2:** Inclusion and exclusion criteria used to select participants for this study.

Inclusion criteria	Exclusion criteria
Male or female	Maxillary anterior teeth with restorations, caries, severe wear, or malposition
Patient aged between 20 and 65 years	Patient with severe gingival inflammation
Patient with 6 maxillary anterior permanent teeth	Presence of primary or missing permanent teeth in the maxillary anterior area
Patient with healthy periodontium	In orthodontic treatment
Informed consent obtained	Not signed consent document

### DICOM segmentation and STL creation

Each patient underwent CBCT (DINNOVA 3; HDX WILL, Seoul, South Korea) scans through the upper anterior part of the maxilla, and the DICOM data were reconstructed to the 3D images with a minimum threshold of 500 Gy values^32^. The radiation dose for CBCT acquisition was 69.0 microsieverts (µSv) with an FOV of 100 × 80 mm at voxel sizes of 0.1 mm^3^. We tried to reduce the scanning time and FOV to minimize image artifacts^31^. By using STL data segmentation software (3D Slicer, Version 4.10.2; Surgical Planning Laboratory, Boston, MA, USA), the anatomies of interest (crown and emergence profile) in the DICOM images were segmented, and then the data were exported to STL files for image processing visualization. The DICOM image segmentation process involved manual threshold selection to determine the region of interest (ROI) from all slices. Processing a large number of triangles would consume a large amount of time and energy. In this study, the data size was reduced to 1.0 mm × 1.0 mm × 1.0 mm when segmenting to STL data.

### Superimposition of 3D datasets

**Figure 4:**
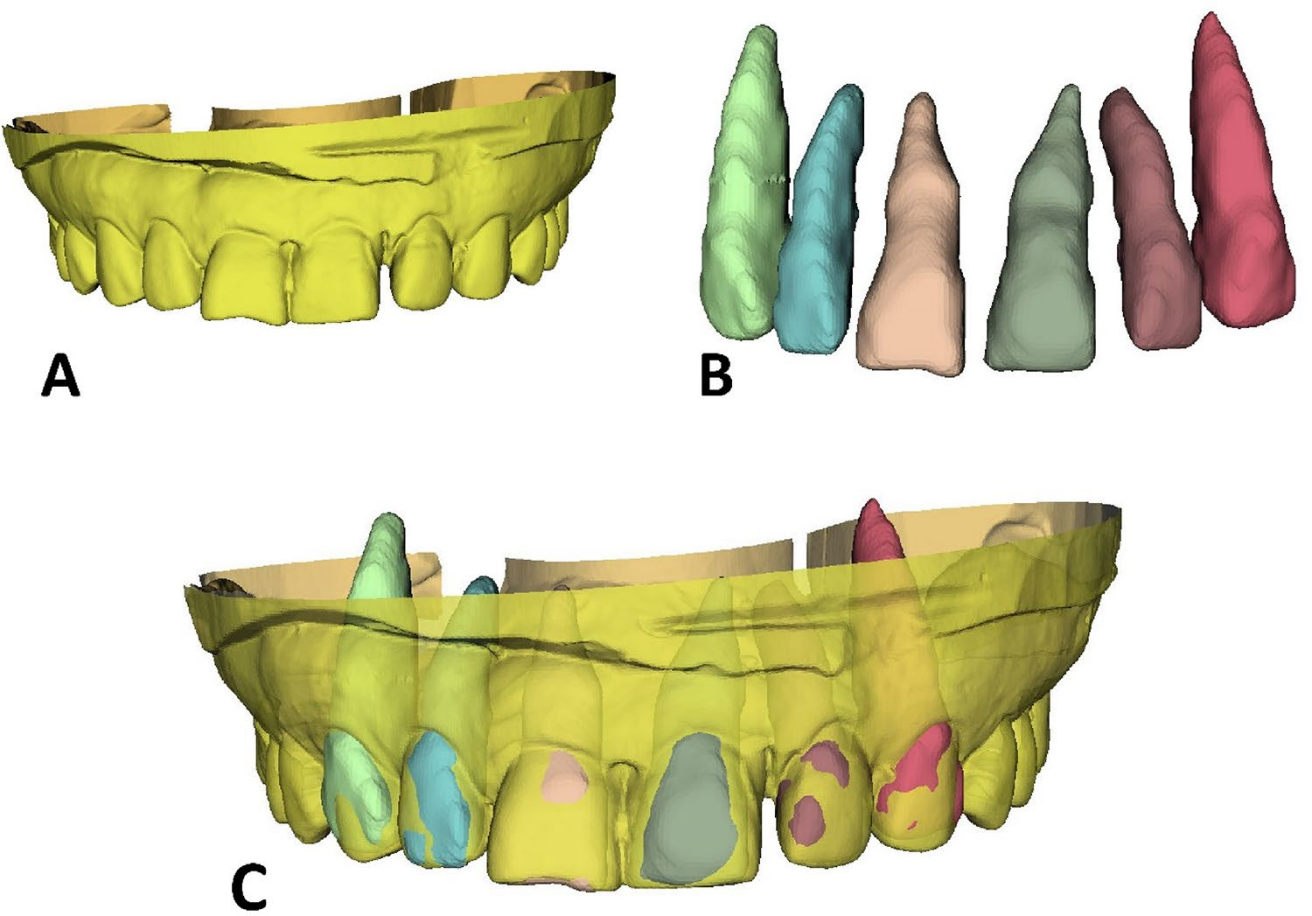
Dataset superimposition. (**A**), A scan image in the STL file format. (**B**), DICOM segmentation and STL conversion. (**C**), Superimposition of two STL files.

**Figure 5:**
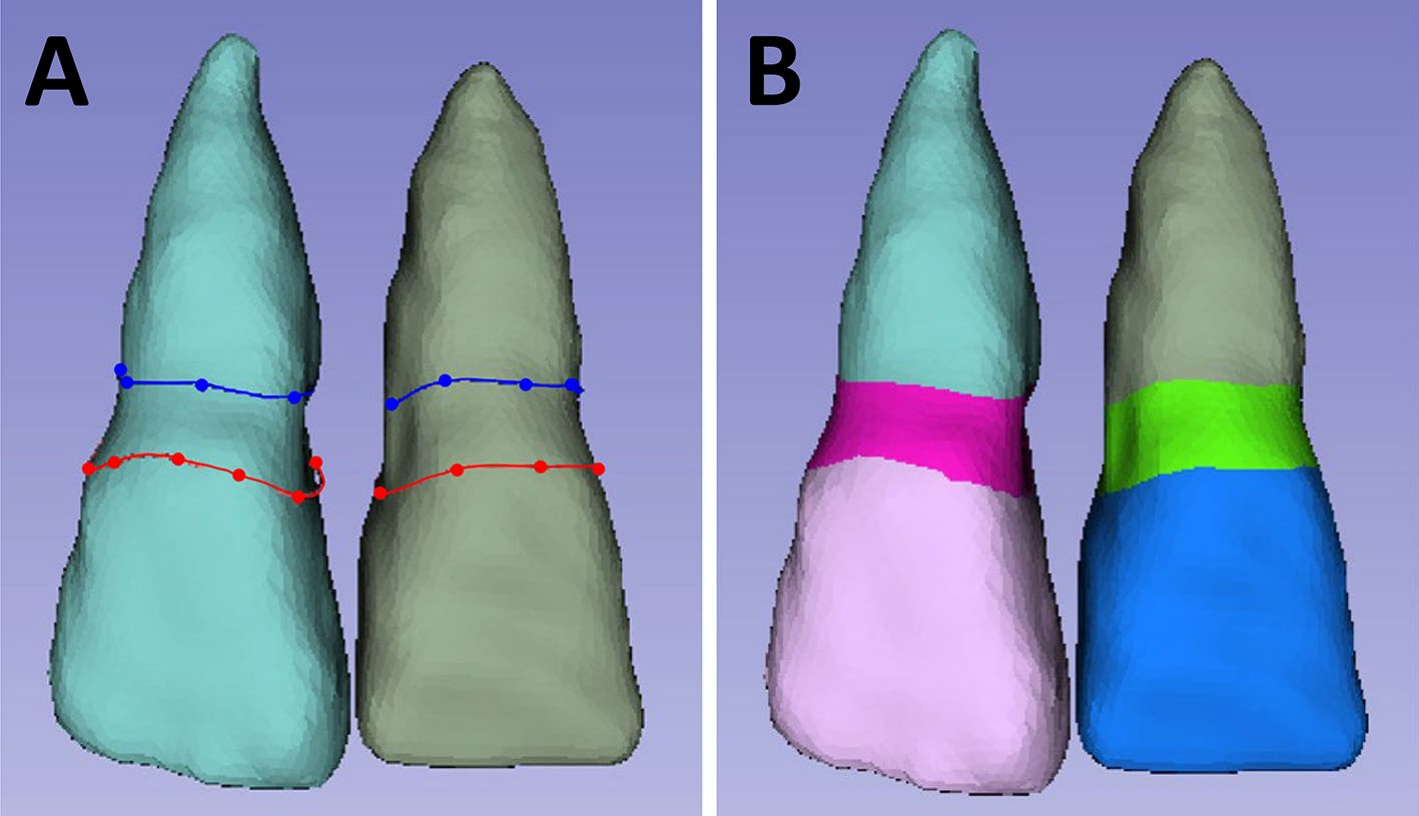
Polygonal annotation of the dataset. (**A**), Detection of the annotated lines. (**B**), Annotation of crown and emergence profile elements.

The individual tooth segments were subsequently superimposed on the STL data acquired by the 3D laboratory scanner through a point-based 3D shape registration technique^33,34^, as shown in Fig. 4. The three reference points used for each superimposition technique were the most apical point of the tooth root and the most prominent point of the incisal edge on the left and right sides. The gingival lines as well as the lines 3 mm below the gingival margin^35^ of the central incisors, lateral incisors, and canines were labeled (Fig. 5A). To determine each annotated line, the geodesic offset line^36^, which is the shortest distance along the surface, and spline interpolation algorithm^37^, which is a special type of piecewise polynomial, were used. Each line was in the form of a closed curve on triangle-based polygonal meshes. Our digital team performed manual annotation with primitive tagging for the region of interest and then, our clinical team corrected and updated the annotation with precise tagging. The annotated datasets included the crowns and the emergence profiles of bilateral central incisors, lateral incisors, and canines (Fig. 5B).

For 3-dimensional image segmentation, a region-based convolutional network (R-CNN)^27,28^, which is a deep learning-based method, was used. In the first stage, the M2Det detection model^38^ produced dense bounding outlines based on the learned features. The second step included multitask learning based on U-Net^39^ with the mask and boundary map of individual elements. All networks were implemented with an Adam optimizer PyTorch technique^40^ at an 1e-4 learning rate. To perform the network, a Nvidia GeForce 2080ti GPU was used.

### Distance calculations

**Figure 6:**
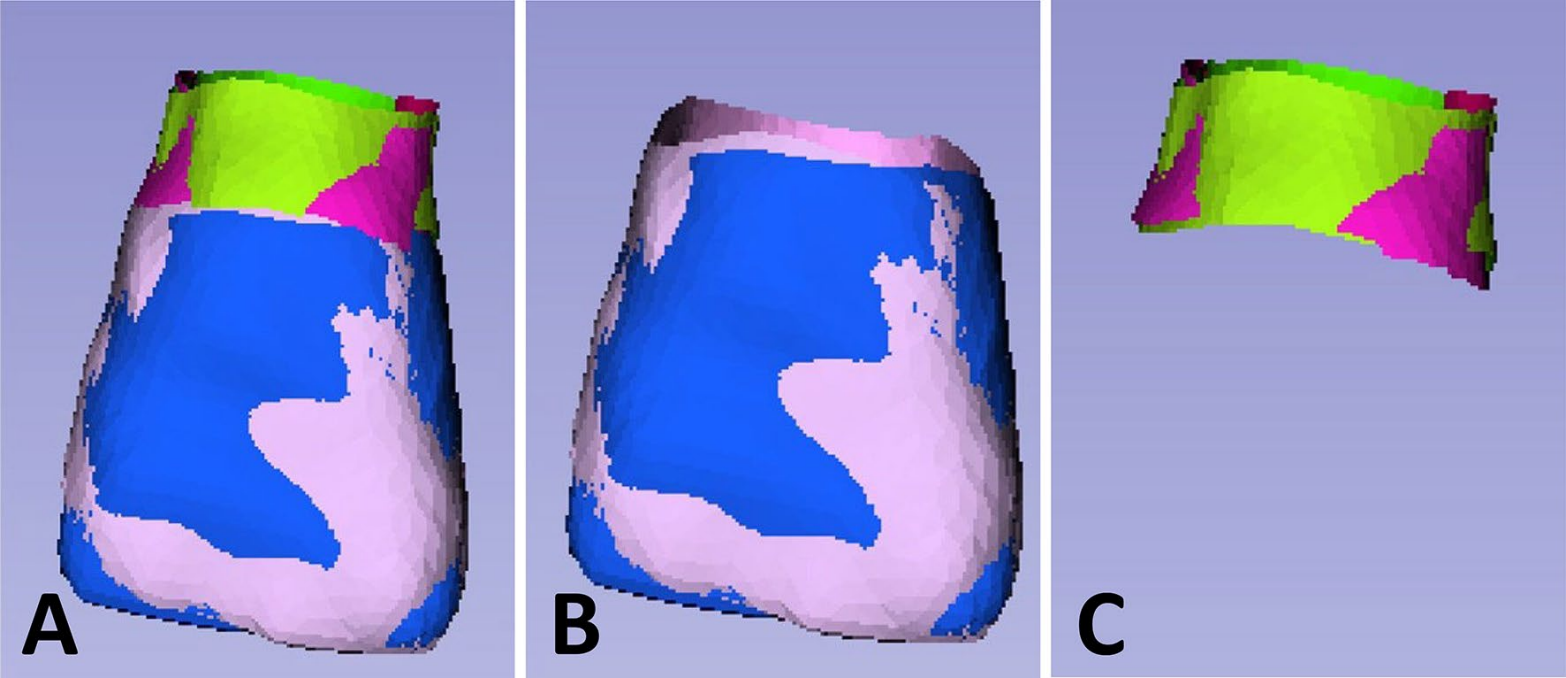
The right element and the left element form a mirrored pair. (**A**), A mirrored pair of crowns with emergence profiles. (**B**), A mirrored pair of crowns. In a mirrored configuration, the blue part indicates one side of the crown, while the pink part indicates the other side of the crown. (**C**), A mirrored pair of emergence profiles. In a mirrored configuration, the green part indicates one side of the emergence profile, while the red part indicates the other side of the emergence profile.

**Figure 7:**
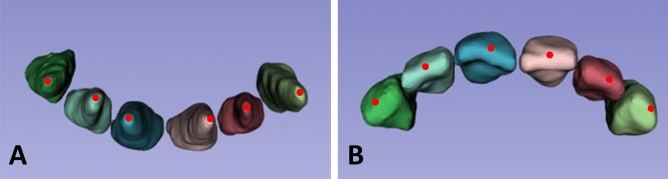
The red dot indicates the apical point (**A**) and the coronal point (**B**) of the long axis.

To evaluate the 3-dimensional shape difference between each pair, the contralateral tooth was digitally flipped (mirrored) along the long axis, and each pair of STL models was superimposed (Fig. 6). With the inclusion–exclusion principle technique, the crown and the emergence profile elements were obtained separately, and the root part was excluded. In this study, pairing was carried out under the assumption that the left-to-mirrored-right pair match agreed with the right-to-mirrored-left pair match. The long axis indicates an imaginary line that passes through the middle point of the crown and the apex of the root (Fig. 7). To register and merge two contralateral images, a constrained iterative closest point (ICP) algorithm^41^ was used, which is a modified point-to-plane ICP to fix the rotation axis and translation plane.

**Figure 8:**
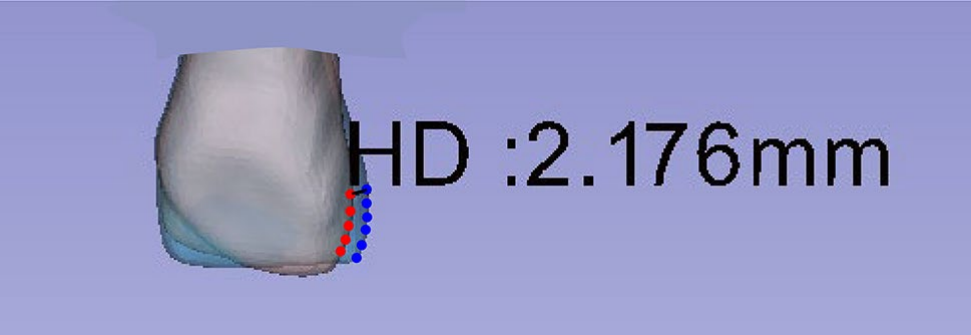
The Hausdorff distance between a pair of crowns. A crown shape must be as close as possible to the other crown shape.

To calculate the overall similarity between the right/left pairs of each element in 3-dimensional space, the HD, which is the longest distance from a point in one set to the closest point in the other set, was measured using MeshLab software (ISTI-CNR, Pisa, Italy)^42^, as shown in Fig. 8. The HD between two point sets of symmetric pairs (right central incisor and left central incisor from the same individual, 100 pairs; right lateral incisor and left lateral incisor from the same individual, 100 pairs; or right canine and left canine from the same individual, 100 pairs) and nonsymmetric pairs (right central incisor and left central incisor from the different individual, 9900 pairs; right lateral incisor and left lateral incisor from the different individual, 9900 pairs; or right canine and left canine from the different individual, 9900 pairs) were calculated for comparing point sets and image segmentations. In this study, the feature point-based matching algorithm proposed by Abdel and Allan^43^ was used to align corresponding features from two similar images.

### Statistics

All statistical analyses were performed using software (SPSS, Version 25.0; IBM, Armonk, NY, USA) with a significance level of 0.05. To evaluate substantial interoperator agreement on specific anatomic locations, Cohen’s kappa (*k*) coefficient was used to measure the strength of agreement between operators. One-way analysis of variance was used to determine whether there was statistical evidence that the associated HD values of true positive pairs, false negative pairs, and true negative pairs were significantly different. The ROC curves were produced to evaluate the performance of the pair comparison algorithm by calculating and plotting the true positive rate (sensitivity) against the false positive rate (1-specificity). To visualize the distribution of overlapping data points of the 3D HD results, jitter plots were created.

## Data Availability

The datasets generated during the current study are available from the corresponding author (H.-K.K.) on reasonable request.
